# NOP2-mediated m5C Modification of c-Myc in an EIF3A-Dependent Manner to Reprogram Glucose Metabolism and Promote Hepatocellular Carcinoma Progression

**DOI:** 10.34133/research.0184

**Published:** 2023-06-30

**Authors:** Hao Zhang, Xiangyu Zhai, Yanfeng Liu, Zhijia Xia, Tong Xia, Gang Du, Huaxin Zhou, Dorothee Franziska Strohmer, Alexandr V. Bazhin, Ziqiang Li, Xianqiang Wang, Bin Jin, Deliang Guo

**Affiliations:** ^1^ Department of Hepatobiliary and Pancreatic Surgery, Zhongnan Hospital of Wuhan University, Wuhan, China.; ^2^ Department of Hepatobiliary Surgery, The Second Hospital of Shandong University, Jinan, China.; ^3^ Organ Transplant Department, Qilu Hospital of Shandong University, Jinan, China.; ^4^ Department of Hepatobiliary Surgery, Qilu Hospital of Shandong University, Jinan, China.; ^5^ Department of General, Visceral, and Transplant Surgery, Ludwig-Maximilians-University Munich, Munich, Germany.; ^6^ Department of Pediatrics Surgery, The Seventh Medical Center of PLA General Hospital, National Engineering Laboratory for Birth Defects Prevention and Control of Key Technology, Beijing Key Laboratory of Pediatric Organ Failure, Beijing, China.

## Abstract

Mitochondrial dysfunction and glycolysis activation are improtant hallmarks of hepatocellular carcinoma (HCC). NOP2 is an S-adenosyl-L-methionine-dependent methyltransferase that regulates the cell cycle and proliferation activities. In this study, found that NOP2 contributes to HCC progression by promoting aerobic glycolysis. Our results revealed that NOP2 was highly expressed in HCC and that it was associated with unfavorable prognosis. NOP2 knockout in combination with sorafenib enhanced sorafenib sensitivity, which, in turn, led to marked tumor growth inhibition. Mechanistically, we identified that NOP2 regulates the c-Myc expression in an m5C-modification manner to promote glycolysis. Moreover, our results revealed that m5C methylation induced c-Myc mRNA degradation in an eukaryotic translation initiation factor 3 subunit A (EIF3A)-dependent manner. In addition, NOP2 was found to increase the expression of the glycolytic genes LDHA, TPI1, PKM2, and ENO1. Furthermore, MYC associated zinc finger protein (MAZ) was identified as the major transcription factor that directly controlled the expression of NOP2 in HCC. Notably, in a patient-derived tumor xenograft (PDX) model, adenovirus-mediated knockout of NOP2 maximized the antitumor effect and prolonged the survival of PDX-bearing mice. Our cumulative findings revealed the novel signaling pathway MAZ/NOP2/c-Myc in HCC and uncovered the important roles of NOP2 and m5C modifications in metabolic reprogramming. Therefore, targeting the MAZ/NOP2/c-Myc signaling pathway is suggested to be a potential therapeutic strategy for the treatment of HCC.

## Introduction

Hepatocellular carcinoma (HCC) is the sixth most common cancer and the fourth most common cause of cancer-related mortality worldwide [1,2]. When diagnosed at an early stage, HCC can be treated with locoregional therapy, which includes surgical resection, radiofrequency ablation, and liver transplantation [3,4]. However, the disease is often diagnosed at an advanced stage, thus rendering many of these treatments ineffective [5]. Therefore, it is essential to identify novel prognostic markers and therapeutic targets for HCC. Despite diagnostic and therapeutic advances, the prognosis of patients with HCC remains dismal. Hence, the underlying molecular mechanisms should be unraveled to develop targeted therapies.

Tumor cells are characterized by increased glucose uptake and adenosine triphosphate generation via glycolysis even under aerobic conditions [6]. Recent studies have revealed that aerobic glycolysis is essential for tumorigenesis and tumor development [7–9]. The process produces large amounts of lipids, proteins, and nucleotides, which accelerate cancer cell proliferation and create an acidic microenvironment that is conducive to cell migration and invasion [10,11].

The field of RNA epitranscriptomics has received increasing attention in recent years [12,13]. At present, over 170 types of RNA modifications have been identified in the mRNA, ribosomal ribonucleic acid, and transfer RNA [14]. These modifications are extremely widespread and functionally modulate the eukaryotic transcriptome to influence RNA splicing, translation, and posttranslational modification [15,16]. Currently, m6A is the most studied RNA modification. Besides m6A, 5-methylcytosine (m5C) is another common mRNA modification. m5C modification plays a key role in tumor and nontumor diseases by participating in various physiological processes [17]. RNA m5C modifications are often linked to epigenetic modifications in the DNA [17,18]. However, the modification also occurs in RNA, mainly transfer RNA and ribosomal ribonucleic acid. This modification can be created or removed by various enzymes that either activate or inhibit the associated signaling pathways. NOP2, a nucleolar RNA-binding protein that contains an RNA-binding domain and an RNA methyltransferase domain, belongs to the m5C methyltransferase family [19].

This study established that NOP2 was obviously up-regulated in HCC and that it was associated with the prognosis of patients with HCC. Subsequently, transcriptomic and metabolomic studies clarified that NOP2 promoted aerobic glycolysis in HCC, which was validated based on glucose uptake, adenosine triphosphate and lactate production, and extracellular acidification rate (ECAR). Furthermore, MYC associated zinc finger protein (MAZ) was identified to be the major transcription factor that directly controlled the expression of NOP2 in HCC. The findings suggested that NOP2 may serve as a novel biomarker and therapeutic target for HCC and demonstrated that the MAZ/NOP2/c-Myc signaling pathway may be a novel mechanism for HCC proliferation and metastasis.

## Results

### NOP2 was up-regulated in HCC and associated with poor prognosis

**Fig. 1.**
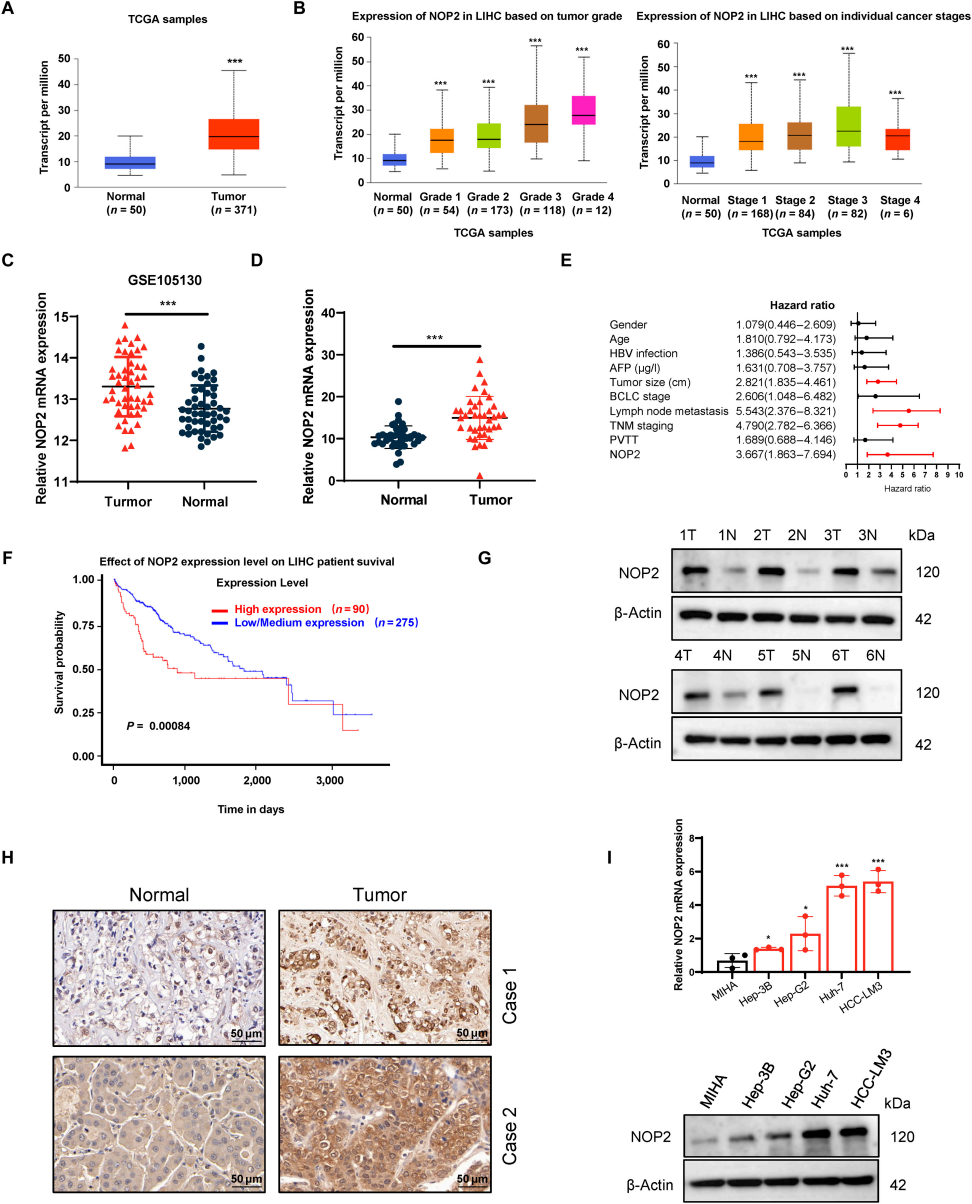
Up-regulation of NOP2 in HCC. (A) NOP2 expression in normal tissues and HCC tissues from TCGA databases. (B) Correlations of NOP2 levels with the tumor stage and grade of HCC. Correlations of NOP2 levels with the tumor stage and tumor grade of HCC from TCGA database. Cancer stage information is not available for 31 samples. Tumor grade information is not available for 14 samples. (C) NOP2 expression in normal tissues and HCC tissues from Gene Expression Omnibus database. (D) NOP2 mRNA expression in liver tissues and HCC tissues. (E) Correlation between NOP2 and clinical pathological parameters of HCC patients. (F) Analysis of overall and relapse-free survival of patients in TCGA dataset. (G and H) NOP2 protein expression in liver tissues and HCC tissues. Scale bars, 50 μm. (I) NOP2 expression in immortalized hepatocytes and HCC cells. *n* = 3 independent experiments. *P < 0.05, **P < 0.01, ***P < 0.001. PVTT, portal vein tumor thrombus.

Analyses of The Cancer Genome Atlas Liver–Hepatocellular Carcinoma (TCGA-LIHC) database showed that NOP2 was highly expressed in HCC and was associated with stage and tumor grade (Fig. 1A and B). Consistently, the Gene Expression Omnibus database (GSE105130) also showed that the expression of NOP2 was markedly increased in HCC tissues (Fig. 1C). The NOP2 expression was determined in 40 pairs of HCC tissues collected from Zhongnan Hospital of Wuhan University with quantitative real-time polymerase chain reaction (qRT-PCR), Western blotting, and immunohistochemical (IHC) analysis. The qRT-PCR results indicated that NOP2 was markedly up-regulated in the HCC tissues and was correlated with higher tumor–node–metastasis (TNM) stage portal vein tumor thrombus, and tumor size (Fig. 1D and Table S1). Univariate and multivariate analysis revealed that NOP2 was an independent factor for overall survival in HCC patients (Fig. 1E and Table S2). Kaplan–Meier survival analyses showed that increased NOP2 expression was statistically associated with poorer overall survival in the TCGA dataset (Fig. 1F). Additionally, Western blotting and IHC analysis verified the high expression of the NOP2 protein in HCC tissues (Fig. 1G and H). Subsequently, the expression of NOP2 was detected in different HCC cell lines. Compared with normal hepatocytes (MIHA), the expression of NOP2 was obviously increased in HCC cell lines (Hep-3B, Hep-G2, Huh-7, and HCC-LM3; Fig. 1I). Collectively, these results suggest that NOP2 may be associated with HCC progression. Hence, NOP2 is expected to be a candidate biomarker for the diagnosis and prognosis of HCC.

### NOP2 promoted in vitro proliferation, migration, and invasion of HCC cells

**Fig. 2.**
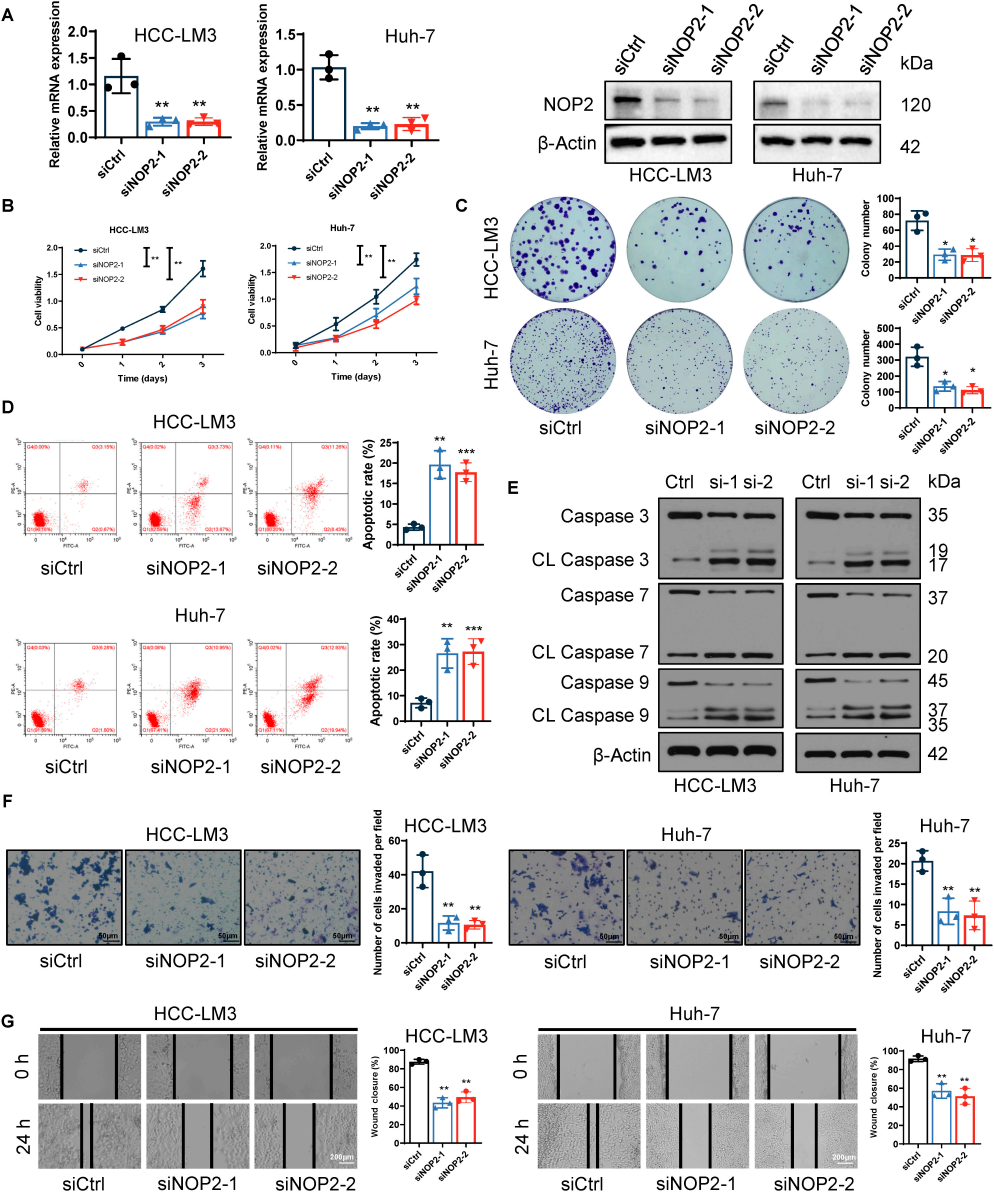
NOP2 promotes HCC cell proliferation and ability of cell invasion and migration. (A) siRNA mediated knockdown efficiency of NOP2. (B and C) Cell proliferation was assessed by performing CCK-8 (OD 450 nm) and colony formation assays. (D) Apoptotic cells were measured using flow cytometry. (E) Western blot assay for apoptotic protein expression. (F and G) Cell invasion and migration were evaluated using wound-healing migration and Transwell invasion assays. Scale bars for Transwell, 50 μm. Scale bars for wound-healing migration, 200 μm. *P < 0.05, **P < 0.01, and ***P < 0.001.

The Huh-7 and HCC-LM3 cell lines with the highest NOP2 expression levels were selected to construct NOP2 knockdown models. Two small interfering RNAs (siRNAs) were designed to construct NOP2 knockdown models, and the knockdown efficiency of each siRNA was confirmed with qRT-PCR and Western blotting (Fig. 2A). The results of Cell Counting Kit-8 (CCK-8) and colony formation assays showed that NOP2 knockdown notably inhibited cell proliferation and colony formation abilities (Fig. 2B and C). Subsequently, the effect of NOP2 on apoptosis was examined in HCC cell lines, and NOP2 knockdown was found to markedly increase the apoptosis rate (Fig. 2D). Furthermore, NOP2 knockdown decreased the levels of caspase 3, 7, and 9, whereas cleaved caspase 3, 7, and 9 were simultaneously increased (Fig. 2E). NOP2 has been reported to substantially regulate cell cycle progression [20]. As expected, NOP2 knockdown greatly increased the HCC cell cycle block (Fig. S1A). The migration and invasion abilities were then determined (Fig. 2F and G). NOP2 knockdown inhibited cell migration and invasion in HCC cells. Furthermore, an overexpression model was constructed using the Hep-3B cell line with the lowest NOP2 expression level (Fig. S2A and B). As expected, CCK-8 and colony formation assays revealed that NOP2 overexpression considerably increased the proliferative capacity of HCC cells (Fig. S2C and D). Moreover, NOP2 overexpression markedly increased the cell invasion and migration abilities of Hep-3B cells (Fig. S2E and F).

### NOP2 promoted in vivo growth and metastasis of HCC cells

**Fig. 3.**
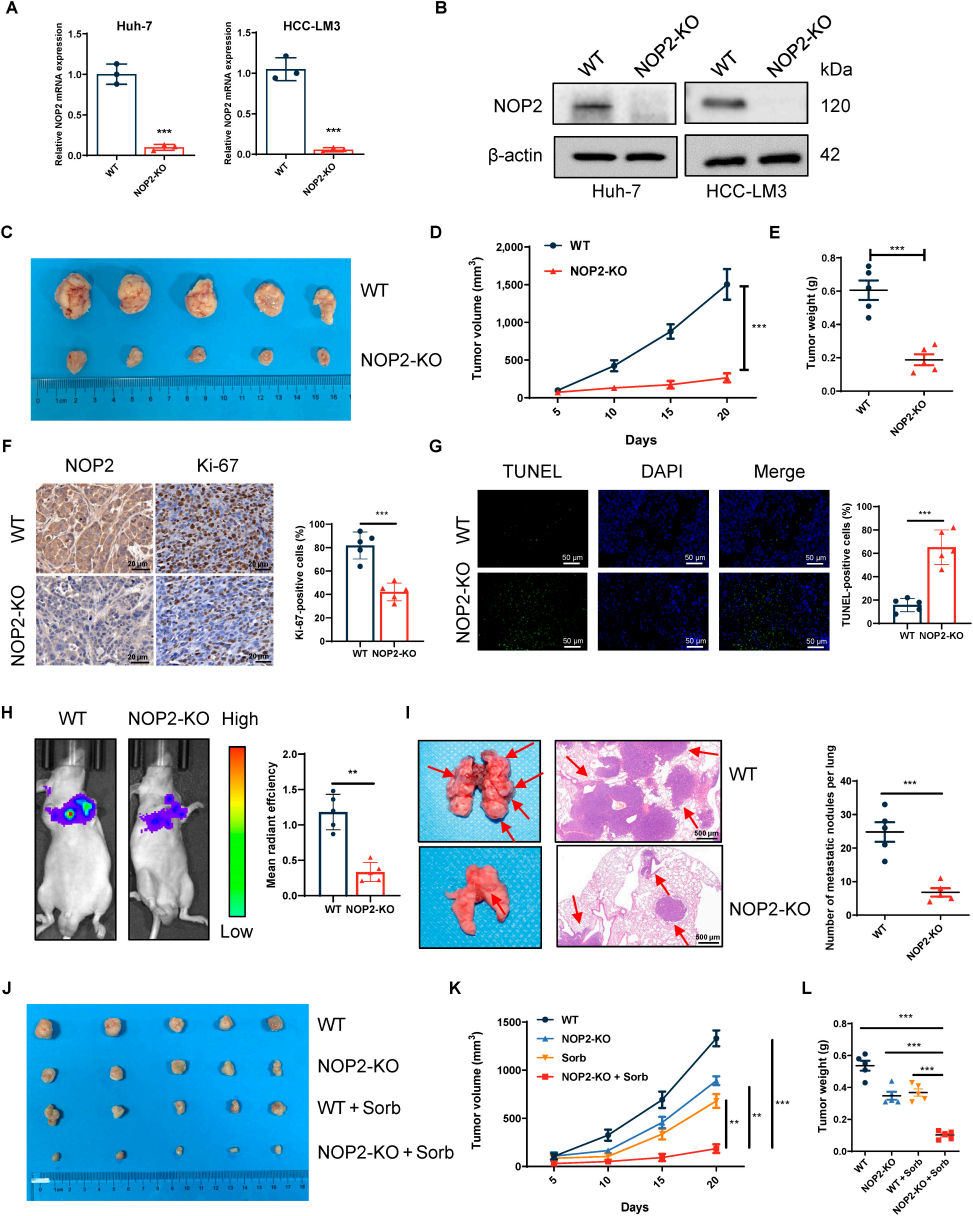
NOP2 promotes HCC growth and metastasis in vivo*.* (A and B) sgRNA mediated KO efficiency of NOP2. (C to E) The volume and growth of subcutaneous xenografts of HCC-LM3 cells. (F) Tumor nodules were subjected to IHC staining for Ki-67 and NOP2. Scale bars, 20 μm. (G) Tumor nodules were subjected to immunofluorescence (IF) staining for TUNEL assays. Scale bars, 50 μm. (H and I) The fluorescence intensity values and the number of metastatic foci were shown. Scale bars, 500 μm. (J to L) The volume and growth of subcutaneous xenografts of HCC cells. *n* = 5 independent experiments. *P < 0.05, **P < 0.01, and ***P < 0.001.

In vivo animal model experiments were performed to examine the effects of NOP2 on tumor proliferation and metastasis. First, NOP2 knockout (KO) cell lines were constructed in HCC cells using the CRISPR/Cas9 system (Fig. 3A and B). Wild-type (WT) and NOP2-KO HCC-LM3 cells were injected subcutaneously into nude mice. Representative gross pictures and tumor growth curves showed that tumor growth of the NOP2-KO group was markedly slower than that of the WT group (Fig. 3C and D). The tumor weight was remarkably decreased in the NOP2-KO group compared with the WT group (Fig. 3E). IHC analysis indicated that compared with the NOP2-KO group, the tumor tissue of the WT group showed higher NOP2 expression (Fig. 3F). In addition, the results of Ki-67 staining signified that compared with the WT group, proliferating cells were markedly reduced in the NOP2-KO group (Fig. 3F). TdT-mediated dUTP nick end labeling (TUNEL) staining results alluded that apoptosis was increased in the NOP2-KO group (Fig. 3G). Subsequently, the experimental lung metastasis model was built in nude mice with tail vein injection of Luc-HCC-LM3 cells (WT or NOP2-KO). The results implied a marked decrease in the number and fluorescence intensity of lung metastatic nodules in HCC cells of the NOP2-KO group (Fig. 3H and I). These results suggest that NOP2 promotes the proliferative and invasive abilities of HCC cells in vivo.

### NOP2 KO in HCC cells notably enhanced the sensitivity to sorafenib

Sorafenib-resistant cells were constructed as reported previously [21]. The role of NOP2 in sorafenib resistance was first explored. Knockdown of NOP2 increased the inhibition rate and decreased the 50% inhibitory concentration values of sorafenib in HCC-LM3R and Huh-7R cells, whereas in cells overexpressing NOP2, the inhibition rate was lower and 50% inhibitory concentrationvalues were higher (Fig. S3A and B). The combination of sorafenib and NOP2 siRNA markedly reduced cell proliferation when compared with siCtrl plus sorafenib (Fig. S3C). Finally, the therapeutic effect of NOP2 KO and sorafenib were validated in vivo. Combination therapy appeared to be more beneficial than sorafenib or NOP2 KO alone (Fig. 3J to L). Taken together, these findings allude that NOP2 KO enhances the antitumor effect of sorafenib and provides new ideas for targeted drug development.

### NOP2 promoted aerobic glycolysis via the up-regulation of glycolytic genes

**Fig. 4.**
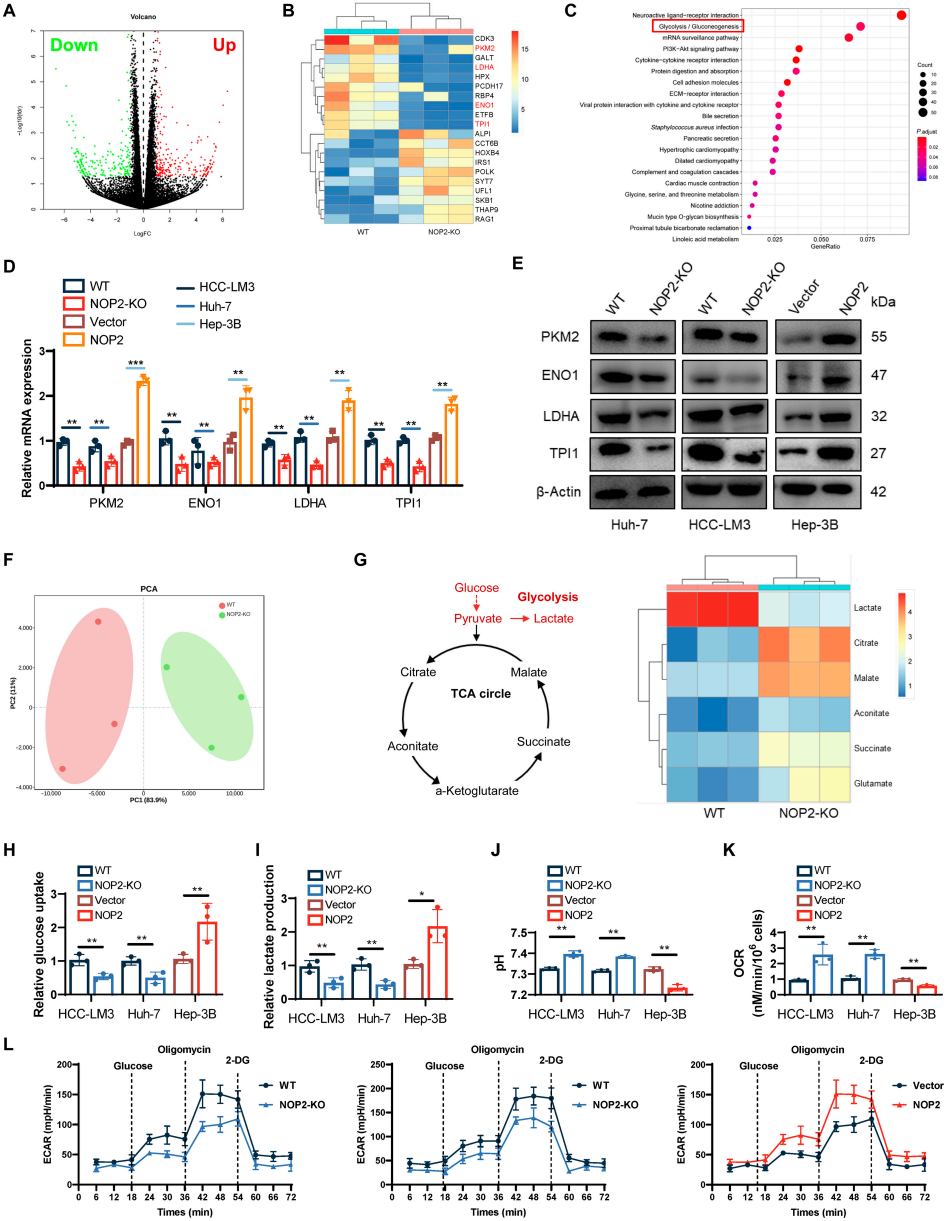
NOP2 enhances the Warburg effect on HCC cells. (A and B) Volcano and heatmaps showing changes in differential genes after NOP2 KO. (C) Kyoto Encyclopedia of Genes and Genomes pathway analysis of RNA-seq data. (D and E) Relative expression levels of proteins related to glycolysis in 3 different cell lines. (F) Nontargeted metabolomics GC-MS analysis of glycolysis and TCA cycle metabolites in HCC-LM3 cells. (G) Heatmap shows changes in metabolites of glycolysis or OXPHOS. (H to K) Glucose uptake (H), lactate production (I), pH of the culture medium (J), and OCR (K) were tested in 3 different cell lines. (L) The ECAR was measured in 3 different cell lines using an XF Extracellular Flux Analyzer. *n* = 3 independent experiments. *P < 0.05, **P < 0.01, ***P < 0.001.

To elucidate the possible molecular mechanism by which NOP2 affects HCC growth and metastasis, differentially expressed genes were screened with RNA sequencing (RNA-seq) after NOP2 KO. The differentially expressed genes between the WT and NOP2-KO groups are shown in the volcano plots and heatmaps (Fig. 4A and B). Kyoto Encyclopedia of Genes and Genomes pathway enrichment analysis indicated that glycolysis was markedly enriched (Fig. 4C). The mRNA and protein expressions of the glycolytic genes PKM2, ENO1, LDHA, and TPI1 were obviously decreased after NOP2 knockdown and markedly increased after its overexpression (Fig. 4D and E). Subsequently, nontargeted metabonomic studies were performed to identify the metabolic pathways affected by NOP2 KO. The principal component analysis demonstrated the relatedness of the samples (Fig. 4F). Heatmap showed the differentially expressed metabolites in the pathways of glucose metabolism (Fig. 4G). The phenotypic changes in aerobic glycolysis after the knockdown of NOP2 were then measured. As expected, silencing of NOP2 markedly reduced glucose uptake and lactate production, whereas its overexpression exerted the opposite effect (Fig. 4H and I). Furthermore, the results indicated that NOP2 knockdown increased the pH and oxygen consumption in HCC cells, whereas its overexpression had the opposite effect (Fig. 4J and K). The ECAR of HCC cells and NOP2 knockdown markedly impaired the glycolytic function of HCC cell lines, whereas overexpression of NOP2 played the opposite role (Fig. 4L). Moreover, the functional specificity of NOP2 KO was rescued by reexpression of NOP2 in KO cells (Fig. S4A and B). The results showed that NOP2 overexpression attenuated NOP2 KO-mediated down-regulation of glucose uptake and lactate production (Fig. S4C and D), up-regulation of pH and oxygen consumption (Fig. S4E and F), and down-regulation of ECAR (Fig. S4G). TCGA database and HCC sample analysis revealed that NOP2 was positively correlated with the glycolytic genes ENO1, LDHA, PKM2, and TPI1 (Fig. S5A and B). These results indicate that NOP2 KO notably reduces aerobic glycolysis in HCC.

### NOP2 regulated c-Myc mRNA m5C methylation and then affected the c-Myc mRNA stability and translation

**Fig. 5.**
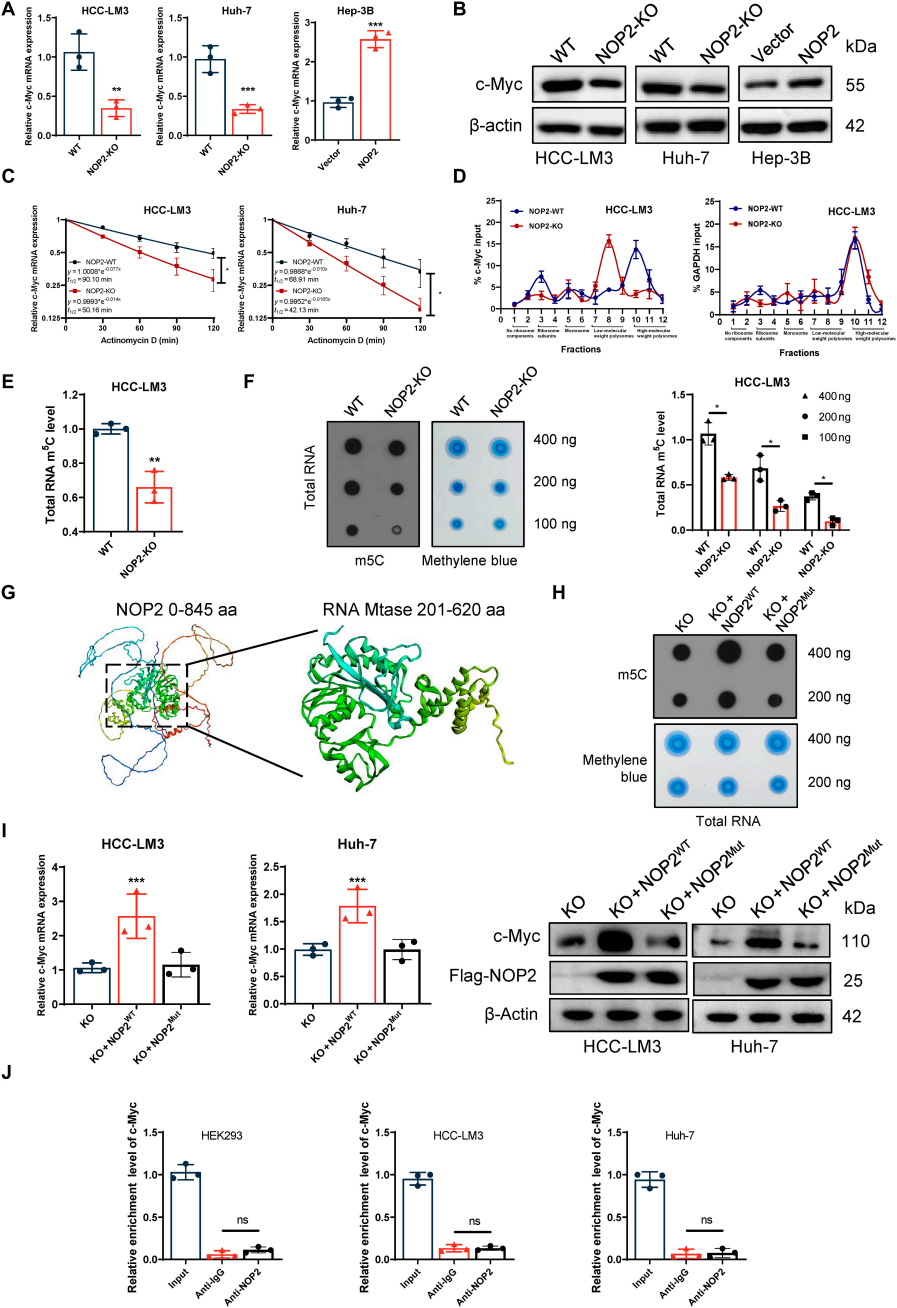
NOP2 regulated c-Myc mRNA m5C methylation and then affected the c-Myc mRNA stability and translation. (A and B) qRT-PCR and Western blot analysis of c-Myc mRNA and protein levels in HCC cells of different treatment groups. (C) qRT-PCR of c-Myc after NOP2 KO followed by actinomycin D treatment (10 μg/ml). (D) The polysomes of NOP2-WT and NOP2-KO cells were extracted and subjected to a 10 to 50% sucrose gradient ultracentrifugation. The mRNA expression level in each fraction was determined by qRT-PCR. (E) LC-MS/MS analysis of m^5^C /C levels in total RNA. (F) Total RNA was used in a dot blot assay (methylene blue staining served as a loading control). (G) Changes of m^5^C level after mutation of NOP2 methylation site. (H and I) Overexpression of NOP2-WT attenuated the effect of NOP2 knockdown in m5C methylation level and c-Myc expression. (J) RIP assays showed that NOP2 is not bound to c-Myc mRNA. GAPDH, glyceraldehyde 3-phosphate dehydrogenase.

The aerobic glycolysis of cancer cells is regulated by several master transcription factors, most notably the c-Myc, P53, and HIF-1a transcription factors [9,22]. The c-Myc levels were observed to decrease after NOP2 knockdown, whereas the levels increased in the case of NOP2 overexpression (Fig. 5A and B). Regretfully, we did not detect any changes in the expression of P53 and HIF-1a. Subsequently, the expressions of c-Myc and NOP2 were measured in 40 pairs of HCC tissue samples. NOP2 and c-Myc were found to be positively correlated at the mRNA and protein levels (Fig. S6A and B). Therefore, c-Myc was investigated as a candidate downstream molecule of NOP2. To confirm that NOP2 regulates the c-Myc expression in HCC cells, we initially examined the transcription and translation of c-Myc when NOP2 knocking out. The results of the actinomycin assay showed that the mRNA decay rate of c-Myc was markedly increased in NOP2-silenced HCC-LM3 and Huh-7 cell lines (Fig. 5C). The results indicated that NOP2 enhanced c-Myc mRNA stability. Sucrose gradient analysis alluded that NOP2 knockdown dramatically decreased the translation of c-Myc but did not alter the translation of the control protein glyceraldehyde 3-phosphate dehydrogenase (Fig. 5D). Based on the methyltransferase profile of NOP2, we speculate that NOP2 regulates c-Myc stability and translation by m5C methylation. Liquid chromatography-mass spectrometry (LC-MS) and dot blot results showed that m5C levels of total RNA were markedly reduced after NOP2 knockdown (Fig. 5E and F). We hypothesized that NOP2 regulate the mRNA stability and translation process of c-Myc by affecting m5C modification. Thus, we constructed a NOP2 mutant without enzymatic activity (Flag-NOP2-MUT) (Fig. 5G). Interestingly, overexpression of Flag-NOP2-WT attenuated the effect of NOP2 knockdown in m5C methylation level and c-Myc expression, but overexpression of Flag-NOP2-MUT did not restore the expression of the c-Myc protein (Fig. 5H and I). Thus, the expression of c-Myc was directly dependent on the RNA methyltransferase activity of NOP2. Finally, to explore whether NOP2 is bound to c-Myc mRNA, we designed primers in c-Myc mRNA and conducted RNA-binding protein immunoprecipitation (RIP) assays. Unfortunately, the data indicated that anti-NOP2 antibodies were not enriched to c-Myc mRNA compared to anti-immunoglobulin G (IgG) antibodies (Fig. 5J).

### The regulation of NOP2 on c-Myc mRNA was EIF3A-dependent

**Fig. 6.**
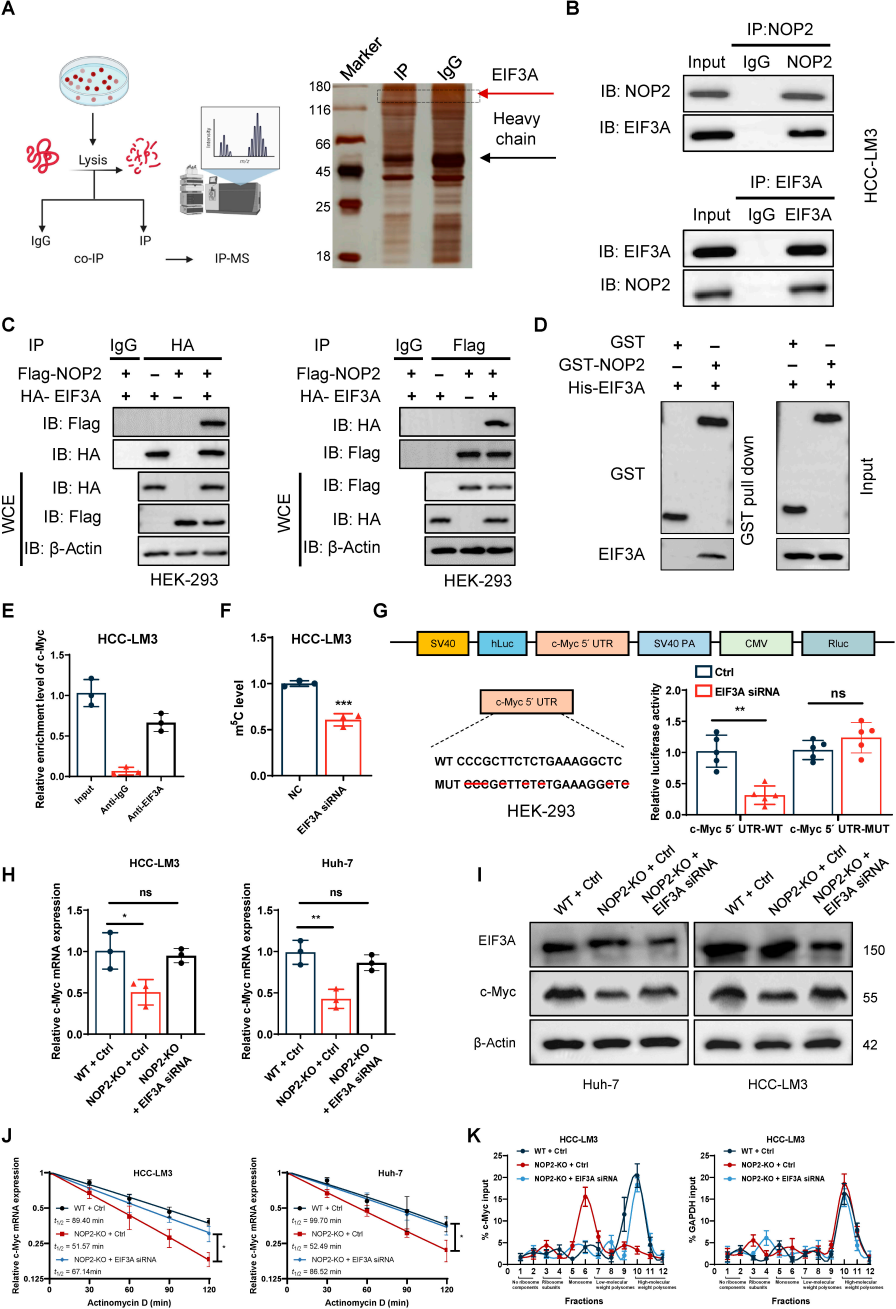
The regulation of NOP2 on c-Myc was EIF3A-dependent. (A) Flow diagram and silver staining showing the IP of NOP2 and subsequent LC-MS/MS analysis. (B and C) Endogenous and exogenous co-IP analysis of the NOP2 and EIF3A interaction. (D) GST pull-down analysis showed direct binding between EIF3A and GST-NOP2. (E) RIP experiment was utilized to verify the binding effect between EIF3A and c-Myc mRNA. The IgG served as a negative control. (F) The changes of m^5^C level in total RNA after knocking down EIF3A. (G) Cells were transfected with c-Myc 5′ UTR-WT or c-Myc 5′ UTR-MUT promoter constructs, and luciferase activity was analysed after transfection. (H and I) qRT-PCR and Western blot analysis of c-Myc mRNA and protein levels in HCC cells of different treatment groups. (J) qRT-PCR of c-Myc after NOP2 KO followed by actinomycin D treatment (10 μg/ml). (K) The polysomes of different treatment groups were extracted and subjected to a 10% to 50% sucrose gradient ultracentrifugation. The mRNA expression level in each fraction was determined by qRT-PCR. *n* = 3 independent experiments. *P < 0.05, **P < 0.01, ***P < 0.001.

To further define the molecular mechanisms by which NOP2 may regulate c-Myc-mediated function in glucose metabolism reprogramming, immunoprecipitation (IP) and liquid chromatography tandem mass spectrometry (LC-MS/MS) analyses were performed to identify the NOP2-bound proteins. Eukaryotic translation initiation factor 3 subunit A (EIF3A) played a central role in maintaining what is often considered the rate-limiting step in mRNA translation [23,24]. Interestingly, the results of silver staining and MS analysis showed that NOP2 interacted with EIF3A (Fig. 6A). This binding was demonstrated using endogenous and exogenous co-IP experiments (Fig. 6B and C). To further corroborate this result, we performed glutathione S-transferase (GST)-receptors pull-down experiments. GST pull-down assay showed that hemagglutinin-EIF3A interacts directly with GST-NOP2, but not to GST (Fig. 6D). This finding suggests that EIF3A may play an important role as a recognizer in NOP2-mediated RNA methylation. To explore whether c-Myc is bound by EIF3A, we designed primers in c-Myc 5′-untranslated region (5′-UTR) and conducted RIP assays. The data indicated that anti-EIF3A antibodies are markedly enriched for c-Myc 5′-UTR compared to anti-IgG antibodies (Fig. 6E). Additionally, LC-MS/MSanalysis exhibited that EIF3A knockdown resulted in a reduction in m5C levels of total RNA in HCC cells. Furthermore, we mutated the m5C site of c-Myc 5′-UTR. Then, human embryonic kidney 293 (HEK293) cells were transfected with mutant 5′-UTR luciferase plasmid and WT 5′-UTR luciferase plasmid. Results of dual-luciferase reporter assay showed that the luciferase activity of WT 5′-UTR decreased after EIF3A silencing, but the luciferase activity of mutant 5′-UTR did not change in HEK293 cells (Fig. 6G). Subsequently, we investigated the effect of EIF3A knockdown on c-Myc expression in NOP2-KO cells. qRT-PCR and Western blotting analysis showed that the regulatory of NOP2 on c-Myc disappeared when knocking down EIF3A (Fig. 6H and I). The results of actinomycin assay showed that the change of mRNA attenuation rate of c-Myc disappeared after silencing EIF3A in NOP2-KO cells (Fig. 6J). The sucrose gradient analysis showed that the translation level changes of c-Myc disappeared after silencing EIF3A in NOP2-KO cell line (Fig. 6K). These results illustrated the regulation of NOP2 on c-Myc was EIF3A-dependent.

### NOP2 promoted HCC growth and metastasis via c-Myc

**Fig. 7.**
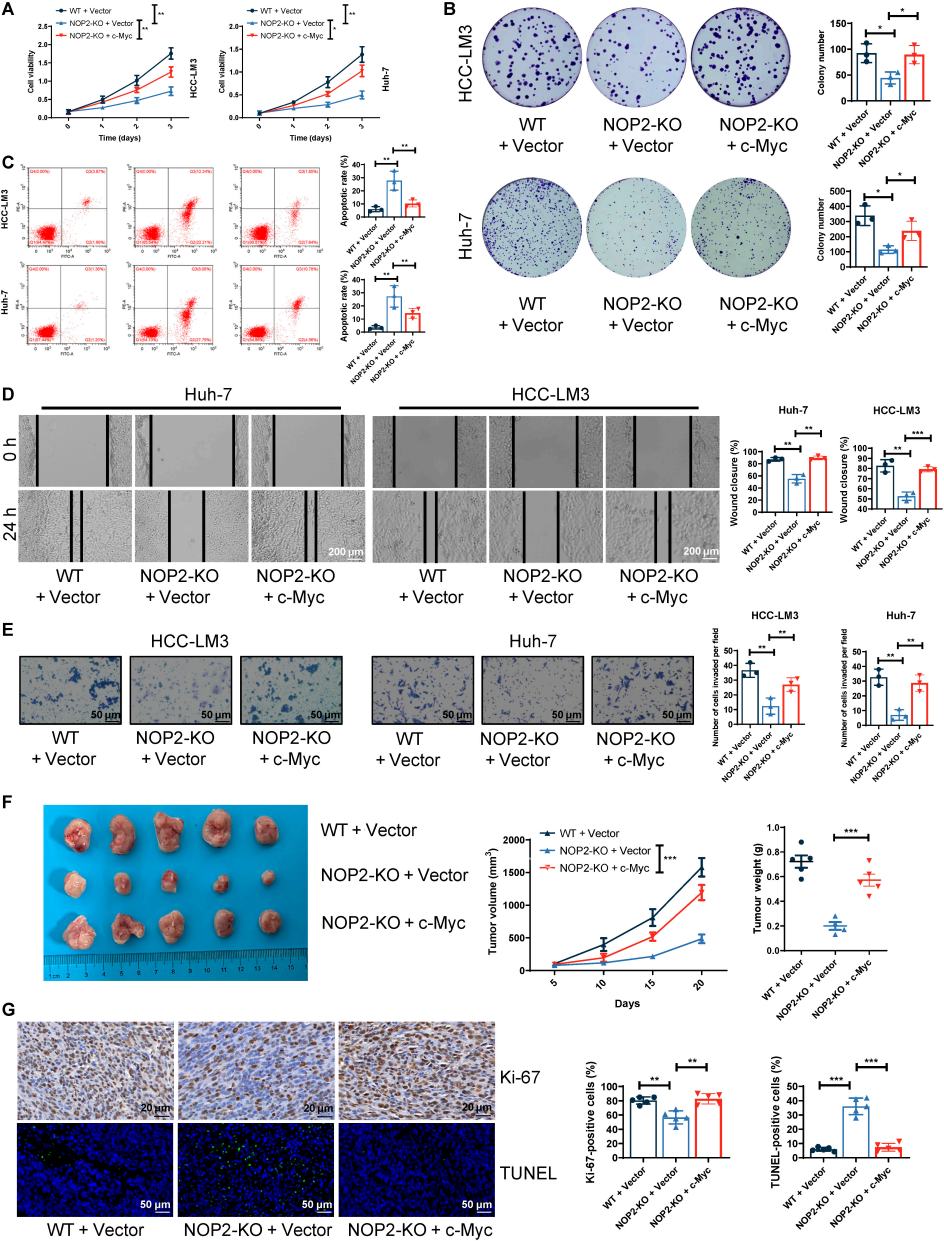
c-Myc plays an essential role in tumor-promoting function. (A and B) Cell proliferation was assessed using CCK-8 (OD 450 nm) and colony formation assays. (C) Apoptotic cells were measured using flow cytometry. (D and E) Cell invasion and migration ability were evaluated using wound-healing migration and Transwell invasion assays. Scale bars for Transwell, 50 μm. Scale bars for wound-healing migration, 200 μm. (F) Curves show the volume and growth of subcutaneous xenografts of HCC cells. (G) Tumor nodules were subjected to IHC staining for Ki-67 and TUNEL assays. Scale bars for Ki-67, 20 μm. Scale bars for TUNEL, 50 μm. For cell experiments, *n* = 3 independent experiments; for animal experiments, *n* = 5 independent experiments; *P < 0.05, **P < 0.01, and ***P < 0.001.

Next, to further investigate whether c-Myc was involved in NOP2-mediated tumor proliferation and metastasis, a rescue assay was performed. CCK-8 and cloning assays showed that the overexpression of c-Myc reversed the inhibitory effect of NOP2 knockdown on cell proliferation (Fig. 7A and B). Results of flow cytometry indicated that c-Myc overexpression rescued the effect of NOP2 knockdown on apoptosis (Fig. 7C). Furthermore, c-Myc overexpression reversed the reduction in invasion and migration abilities caused by NOP2 silencing (Fig. 7D and E). Moreover, the effect of c-Myc overexpression on the NOP2-mediated aerobic glycolytic phenotype was explored. The results showed that c-Myc overexpression attenuated NOP2 knockdown-mediated down-regulation of glycolytic genes (Fig. S7A and B), down-regulation of glucose uptake and lactate production, up-regulation of pH and oxygen consumption (Fig. S8A), and down-regulation of ECAR (Fig. S8B). Tumor xenograft in nude mice revealed that c-Myc overexpression partly rescued the inhibitory effects of NOP2 knockdown on the growth rate in vivo (Fig. 7F). Ki67 and TUNEL staining results showed that c-Myc overexpression partially rescued the effects of NOP2 knockdown on cell proliferation and apoptosis in vivo (Fig. 7G). Taken together, the results strongly suggest that c-Myc promotes tumor progression by acting as a downstream target of NOP2.

### MAZ activated the transcription of NOP2

**Fig. 8.**
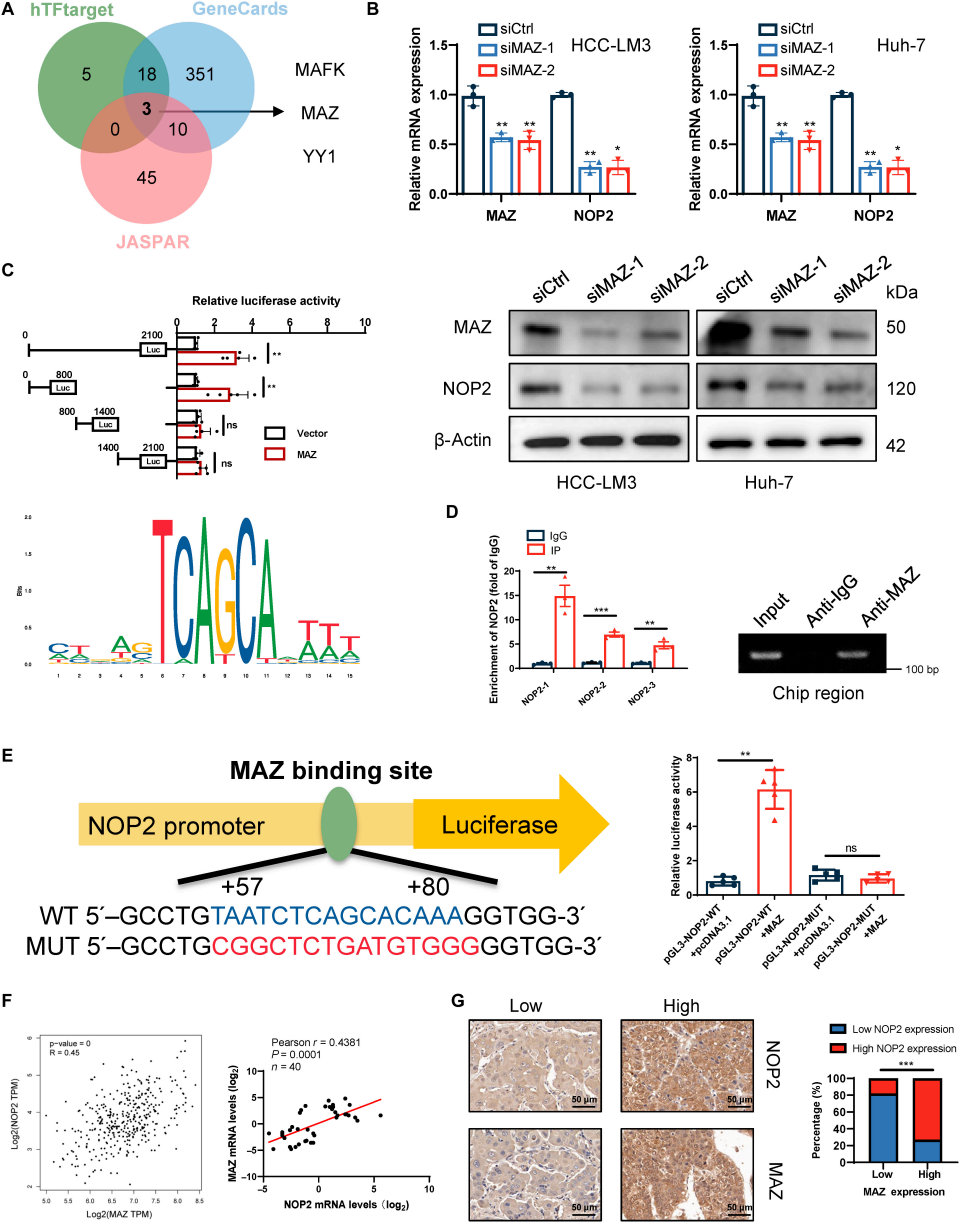
MAZ promotes NOP2 transcription. (A) Venn diagram showing transcription factors that bind the promoter region of NOP2 identified in the GeneCards, JASPAR, and TFtarget databases. (B) Effect of MAZ knockdown on NOP2 protein expression. (C) Schematic of the putative MAZ binding motif and relative score determined using JASPAR. Cells were transfected with the full-length NOP2 promoter or 1 of 3 truncation mutants, and the luciferase activity was analyzed after transfection. (D) ChIP analysis of MAZ binding to the NOP2 promoter. The input and IgG served as positive and negative controls, respectively. (E) Cells were transfected with NOP2-WT or NOP2-MUT promoter constructs, and luciferase activity was analyzed after transfection. (F and G) Correlation analysis of NOP2 and MAZ expression in HCC tissues. Scale bars, 50 μm. *n* = 3 independent experiments; *P < 0.05, **P < 0.01, and ***P < 0.001.

The upstream transcription factors of NOP2 were screened with GeneCards, hTFtarget, and JASPAR databases (Fig. 8A). The results of Western blotting and qRT-PCR confirmed that MAZ knockdown down-regulated the NOP2 expression in HCC cells, whereas MAFK or YY1 knockdown resulted in no marked alteration in NOP2 expression (Fig. 8B). Therefore, MAZ was identified as the main transcription factor of NOP2 for further studies. NOP2 promoter region and promoter truncations were cloned and fused to a luciferase reporter. Dual luciferase reporters indicated the binding of MAZ to the NOP2 0-800 promoter region (Fig. 8C). Then, the DNA motif of NOP2 was obtained from JASPAR. Chromatin IP (ChIP) assays were performed, which confirmed that MAZ binds to the 0-800 region of the NOP2 promoter (Fig. 8D). Luciferase reporter vectors containing WT or mutant NOP2 binding sequences were constructed. The results showed that the mutation of the binding site abolished the ability of MAZ to promote NOP2 expression (Fig. 8E). The findings further revealed a positive correlation between MAZ and NOP2 in clinical samples (Fig. 8F and G). Collectively, the transcription factor MAZ binds to the NOP2 promoter region and promotes its transcription.

### NOP2 KO inhibited HCC growth in the patient-derived tumor xenograft model

**Fig. 9.**
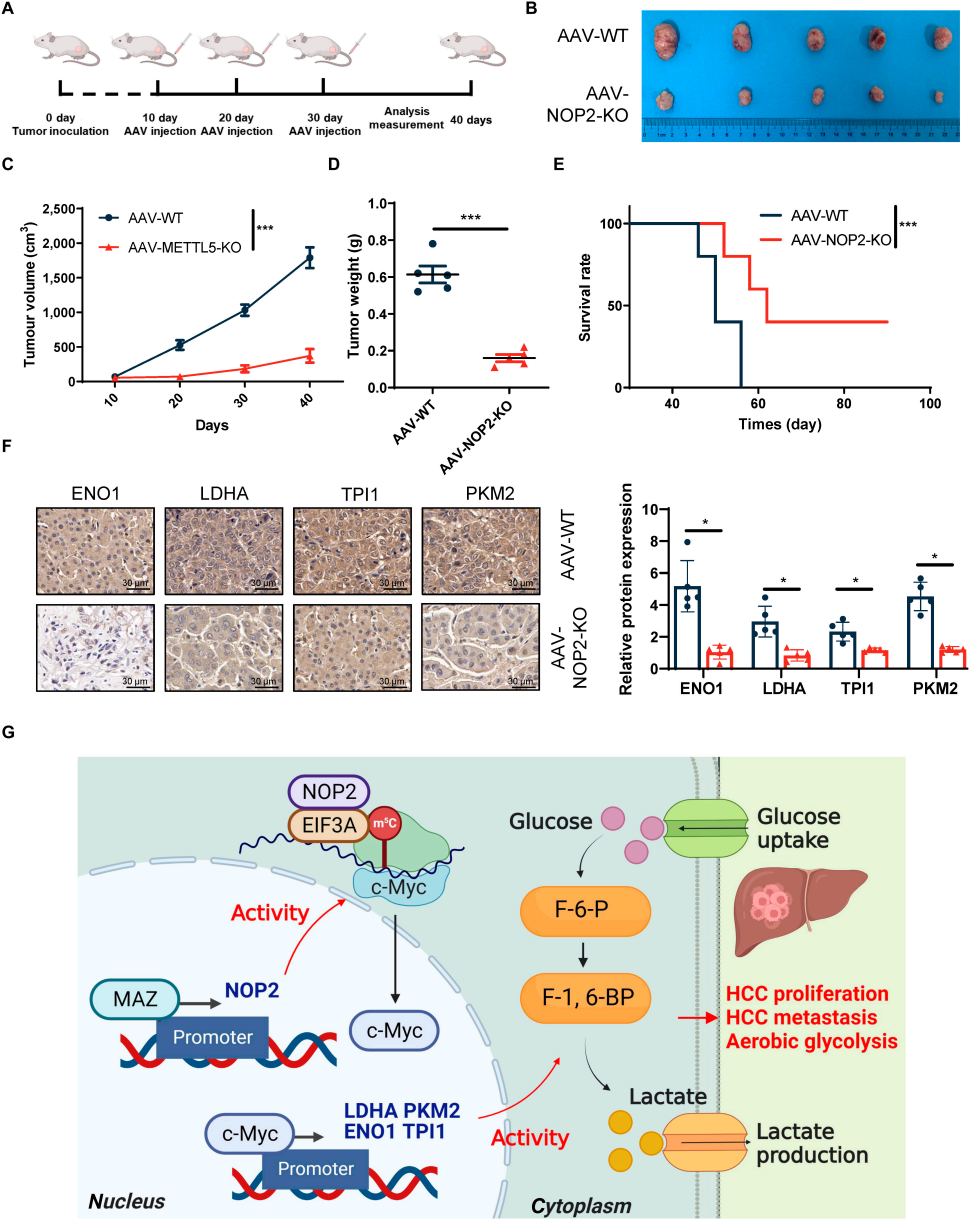
Marked antitumor effects of NOP2-KO in HCC PDX models. (A) Schematic representation of the treatment course. (B to D) Tumor volume and growth curves of PDX model. (E) Survival curves of PDX mice treated in AAV-WT or AAV-NOP2-KO. (F) Tumor nodules were subjected to IHC for Ki-67, IF staining for TUNEL. Scale bars, 50 μm. (G) Schematic diagram showing that NOP2 promotes c-Myc expression in an m^5^C-dependent manner. *n* = 5 independent experiments; *P < 0.05, **P < 0.01, and ***P < 0.001.

Patient-derived tumor xenograft (PDX) models are considered accurate and reliable preclinical models because they resemble the patients’ original clinical cancer [25]. Fresh HCC patient samples were implanted into NCG mice to determine whether NOP2 KO exerted a comparable effect on the PDX model (Fig. 9A). Adeno-associated virus (AAV)-mediated NOP2 KO demonstrated a marked antitumor effect (Fig. 9B to D and Fig. S9A). NOP2 KO efficiency was testified by IHC (Fig. S9B). Furthermore, NOP2 knockdown notably increased the survival time of the PDX model (Fig. 9E). The body weights of the mice did not differ markedly during the treatment period (Fig. S9C). The AAV-NOP2-KO group exhibited lower ENO1, LDHA, TPI1, and PKM2 expressions (Fig. 9F). The excellent tumor suppressor effect of NOP2 KO in PDX models indicates the immense potential of NOP2 activity inhibition in clinical therapeutic development.

## Discussion

Altered cell metabolism is one of the hallmarks of cancer, and aerobic glycolysis has been observed in different types of human tumors [6,26]. The aerobic glycolysis (Warburg effect) was firstly reported in rat liver carcinoma in the 1920s, compared with normal cells, cancer cells use aerobic glycolysis as their primary energy source [7,27]. Various metabolites from aerobic glycolysis are available for biosynthesis to meet the needs of fast-growing tumors [10]. Furthermore, most studies have shown that multiple glycolytic enzymes are overexpressed in several human carcinomas and are linked to poor outcomes. For example, LDHA is highly expressed in various cancers, and its knockdown notably inhibits tumor growth [28]. PKM2, which converts phosphoenolpyruvate to pyruvate, also plays a key role in the progression of cancer [29,30]. In addition, lactate production creates an acidic environment that aids in cancer invasion and metastasis [31]. Furthermore, numerous studies have demonstrated that increased aerobic glycolysis is associated with sorafenib resistance. Aerobic glycolysis plays an important role in the proliferation, growth, invasion, and treatment of cancer [7]. Deeper insight into the role of aerobic glycolysis in HCC will provide valuable information regarding pathogenesis and potential treatment options for HCC, as well as unlocking the mechanism of resistance to sorafenib.

Previous studies have shown that NOP2 was notably up-regulated in most cancers, and high NOP2 expression was associated with poor prognosis [32]. Notably, the role of NOP2 in the reprogramming of tumor glucose metabolism has not been reported. Our study has established the pertinent role of NOP2 in regulating glucose metabolism in HCC. NOP2 was found to be highly expressed in HCC, and patients with a high NOP2 expression exhibited poor prognoses. High expression of NOP2 obviously promoted aerobic glycolysis and proliferation of HCC. Sorafenib is a multikinase inhibitor approved for the first-line systematic treatment of HCC [33]. However, the drug benefits only 30% of the patients; moreover, even those patients who are sensitive to the drug develop resistance within 6 months [34]. Recently, studies have reported that sorafenib can promote aerobic glycolysis in HCC [35,36]. Our findings demonstrated the beneficial effect of NOP2 inhibitors on tumor growth in patients with HCC and indicated the possibility of using them in combination with sorafenib, thereby facilitating the development of anti-HCC drugs and therapeutic strategies in the future.

The oncoprotein c-Myc activates almost all glycolytic genes and plays an essential role in regulating glycolysis under normoxic conditions [22]. Combined analysis of RNA-seq and metabolomic data identified c-Myc as a downstream molecular of NOP2. In vivo and in vitro assays revealed that NOP2 enhanced c-Myc expression and that the suppressive effect of NOP2 was partially rescued by c-Myc overexpression. Additionally, NOP2 regulated PKH2, LDHA, TPI1, and ENO1 via c-Myc. These findings suggest that the NOP2-c-Myc axis plays a critical role in the glycolytic transition.

m5C regulates various cellular processes, including cell self-renewal, differentiation, invasion, and apoptosis [37]. m5C is associated with various cellular processes and systemic diseases, including cell proliferation and cancer metastasis [18]. Regulators of m5C can be broadly classified into 3 types: writers, erasers, and readers. Writers (methyltransferases), catalyze the formation of m5C. Erasers (demethylases), remove m5C from RNA. Readers are RNA-binding proteins that recognize m5C and execute corresponding functions [38]. For example, the m5C methyltransferase NSUN2 promotes the proliferation, migration, and invasion of gastric cancer cells [39]. NSUN2 and YBX1 drive the pathogenesis of urothelial carcinoma of the bladder by targeting the m5C methylation site in the 3′-untranslated region of HDGF [40]. Although the roles of m5C have been reported previously, the scientific community’s understanding of the underlying mechanisms in m5C modification is far from complete. Our results showed that NOP2 regulates the stability and translation of c-Myc in an m5C-dependent manner, thereby promoting aerobic glycolysis. Furthermore, our data provided clear evidence that NOP2-mediated m5C modification and degradation of c-Myc mRNA are EIF3A dependent. Our results showed that EIF3A bound to c-Myc mRNA, whereas NOP2 does not. Moreover, we also found that NOP2 bound to EIF3A, and NOP2 catalyzed m5C modification of c-Myc mRNA only in the presence of EIF3A. These suggested that EIF3A might play a chaperonin role as a reader of NOP2 (m5C binding protein), but this needs further EIF3A protein structural experiments to verify.

MAZ is a transcription factor that participates in transcription initiation and termination [41]. MAZ is highly expressed in many human tumors and promotes cancer development, progression, and metastasis via transcriptional activation of multiple downstream target genes [42]. Our study indicated that MAZ can activate NOP2 transcription by binding directly to its promoter region. In the TCGA database and clinical samples, a markedly positive correlation exists between the expressions of MAZ and NOP2.

Our results revealed a novel regulatory mechanism wherein NOP2 is transcriptionally activated by MAZ and regulates m5C modification, stability, and translation of c-Myc mRNA, thereby promoting HCC proliferation and metastasis (Fig. 9F). Our findings provide novel insights into NOP2-mediated aerobic glycolysis and facilitate the development of novel therapeutic strategies and drugs in the future. However, the use of the abnormal glucose metabolism in HCC cells as an early diagnostic indicator of liver cancer and its further application in targeted therapy are major issues to be tackled by the researchers.

## Materials and Methods

### Clinical specimens and cell culture

A total of 40 HCC and paraneoplastic tissues were obtained from patient volunteers who had their tumors removed at the Wuhan University Central South Hospital and Qilu Hospital of Shandong University. All patients provided their written informed consent. All tumor samples were histopathologically verified as HCC by 2 senior independent pathologists. This study was conducted in accordance with the Declaration of Helsinki and approved by the Ethics Committee of Wuhan University Zhongnan Hospital. HEK293T, Huh-7, HCC-LM3, Hep-3B, and Hep-G2 cells were purchased by the Stem Cell Bank, Chinese Academy of Sciences (Shanghai, China). All cell lines were verified by short tandem repeat profiling and routinely tested for mycoplasma contamination. All cells were cultured in a 37 °C, 5% CO_2_ incubator. HEK293T, Huh-7, HCC-LM3, and Hep-G2 cells were cultured in the Dulbecco's modified Eagle medium (Gibco, Carlsbad, CA, USA) supplemented with 10% fetal bovine serum (BD Bioscience, San Jose, CA, USA). Hep-3B cells were cultured in the minimum essential medium (Gibco) supplemented with 10% fetal bovine serum, 1% GlutaMAX (Gibco), 1% nonessential amino acids (Gibco), and 100 mM solution of 1% sodium pyruvate (Gibco).

### RNA extraction and qRT-PCR

Total RNA was extracted with TRIzol (Invitrogen, USA). qRT-PCR was performed in accordance with the manufacturer’s instruction of Chamq Universal Sybr Qpcr Master Mix (Vazyme, Nanjing, China). The sequences of primers are listed in Table S3.

### Immunohistochemistry and immunofluorescence staining

Immunohistochemistry and immunofluorescence staining were performed as previously reported [43]. The staining intensity was scored as follows: colorless (no staining) was 0 points, light yellow (weak staining) was 1 point, yellow-brown (moderate staining) was 2 points, and brown (strong staining) was 3 points. The percentage of positive cells was scored as follows: ≤25% positive cells were scored as 1 point, between 26% and 50% positive cells were scored as 2 points, between 51% and 75% positive cells were scored as 3 points, and >75% positive cells were scored as 4 points; Intensity of immunoreactivity(IRS) = staining intensity × positive rate. In the final analysis, IRSs ≤ 6 were defined as low expression, while IRSs > 6 were defined as high expression.

### Western blot analysis

Western blot and co-IP assays were performed as described previously [44]. All antibodies used in the present study is presented in Table S4.

### CRISPR/Cas9

Single guide RNA (sgRNA) was designed and cloned following the general cloning protocol of Feng Zhang’s lab (http://crispr.mit.edu). Briefly, sgRNA was inserted into the lentiCRISPR V2 vector (Addgene). lentiCRISPRv2-sgRNA NOP2 and scramble transfer plasmids were used, and the packaging plasmids pMD2.G and psPAX2 (Addgene) were cotransfected. After the addition of polyethylene, infection was performed with a viral supernatant. The knockdown efficiency was determined by qRT-PCR.

### Cell transfection

For NOP2 knockdown, siRNA was designed by the GenePharma Company (Shanghai, China). Transfection was conducted with the Lipo3000 in accordance with the manufacturer’s instructions. For the overexpression of NOP2, NOP2 was subcloned into the pcDNATM3.1 vector.

### CCK-8 assay and colony-formation assay

CCK-8 was determined in accordance with the manufacturer’s protocol (HY-K0301, Shanghai, China). The colony-forming activity was measured according to the established procedure. Briefly, 1,000 cells were cultured for 14 d, after which the cells were fixed with 4% paraformaldehyde for 30 min and stained with 0.5% crystal violet for 60 min. The cell colonies were counted and stained, and the photos were analyzed.

### Scratch wound-healing motility assay and Transwell invasion assay

Scratch wound-healing motility assays, and Transwell invasion assays were performed as described previously [45].

### Apoptosis analysis and cell cycle analysis

The Annexin V-FITC/PI Apoptosis Detection Kit (Lianke, Hangzhou, China) was used to detect apoptosis and analyze the cell cycle. After staining, the cells were analyzed by flow cytometry (BD Bioscience Company, San Jose, California). Flowjo 7.2.5 software (American Flowjo LLP) was used to analyze the data.

### In vivo experiments

All mice studies were approved by the Animal Ethics and Welfare Committee of Central South Hospital of Wuhan University. Four-week-old female BALB/c nude mice were purchased from Jichui Yao Kang Biotechnology Co., Ltd. (Nanjing, China). For the mouse experiment, the animals were randomly assigned to experimental groups of 5 mice. Control or stable NOP2-KO HCC-LM3 cells were subcutaneously injected into the axillary or tail vein of BALB/c nude mice. Four weeks after the cell injection, the mice were killed, their tumors were collected, and their weights were measured, followed by the removal of their lungs for HE staining. For the PDX model, the HCC tissues were quickly cut into small pieces of 3 × 3 × 3 mm on an ice bath and then inoculated into the right forelimb of the NCG mice. When these tumors reached a volume of approximately 200 mm^3^, the mice were randomly assigned to 2 groups for antitumor research and survival evaluation.

### Measurement of the ECARs and the oxygen consumption rate

ECAR and oxygen consumption rate (OCR) were analyzed by the XFe96 Extracellular Flow Analyzer (Seahorse Bioscience, North Billerica, USA). Briefly, 1 × 105 cells/well were inoculated in an XF96-coated cell-culture microplate. Then, the cells were incubated in a medium supplemented with 2 mM glutamine, 10 mM glucose, 1 mM pyruvic acid, and 5 mM HEPES for 45 min. Finally, ECAR and OCR were monitored as recommended by the manufacturer.

### Dual-luciferase assay

A double luciferase assay kit (Promega, Madison, USA) was used for double luciferase assay. WT and mutant NOP2 were inserted into the pGl3 basic vector plasmid. Lipofectamine 3000 (Thermo Fisher) was used for transient transfection. The luciferase activity was measured by the double luciferase assay system (Promega). The renal luciferase activity was assessed to standardize the firefly luciferase activity.

### ChIP assay

The Chip Detection Kit (Thermo) was used for chip detection. The sample was cross-linked with 1% formaldehyde and subjected to ultrasonic treatment to obtain a DNA fragment of size 200 to 500 bp. Then, 2 μg anti-NOP2 or anti-IgG antibody was used to immunoprecipitate the samples treated by ultrasound. qRT-PCR analysis was performed after elution, protease K treatment, and cross-linking reversal.

### Quantification of RNA modification (LC/MS-MS)

UPLC-ESI-MS/MS system (UPLC, ExionLC AD; MS, Applied Biosystems 6500 Triple Quadrupole) was used to detect m5C/C level. The analytical conditions were as follows: LC: column, Waters ACQUITY UPLC HSS T3 C18 (1.8 μm, 2.1 mm*100 mm); solvent system, water (2mM NH_4_HCO_3_): methanol (2mM NH_4_HCO_3_); gradient program, 95:5 v/v at 0 min, 95:5 v/v at 1 min, 5:95 v/v at 9 min, 5:95 v/v at 11 min, 95:5 v/v at 11.1 min, 95:5 v/v at 14 min; flow rate, 0.30 ml/min; temperature, 40°C; injection volume: 10 μl. The effluent was alternatively connected to an ESI-triple quadrupole-linear ion trap-MS. After the MS data of different samples are obtained, the chromatographic peaks of all the targets are integrated, and quantitative analysis is carried out by standard curve.

### Statistical analyses

SPSS software (version 22.0, SPSS, Chicago, IL) was used for all statistical analyses. Student *t* test was performed to compare the 2 groups of data. Spearman’s coefficient was used to evaluate the correlation between the variables. Unless otherwise stated, all experiments were repeated 3 to 5 times independently. *P* ≤ 0.05 was considered to indicate statistical significance.

## Data Availability

All the data needed to evaluate the conclusions in the paper are present in the paper and in the Supplementary Materials. Additional data related to this paper may be requested from the authors.

## Supplementary Materials

Supplementary 1 Fig. S1. (A) Flow cytometry results showing changes in cell cycle progression. Fig. S2. (A and B) The NOP2 overexpressing plasmid was transformed into the Hep-3B cell line. (C and D) Cell proliferation was assessed by performing CCK-8 (OD 450 nm) and colony formation. (E) Cell invasion was evaluated using Transwell invasion assay. (F) Cell migration was evaluated using wound-healing migration. Fig. S3. (A) The effect of NOP2 knockdown on sorafenib resistance. (B) The effect of NOP2 overexpression on sorafenib resistance. (C) CCK-8 assay results showing the proliferation ability of HCC cells. Fig. S4. (A and B) qRT-PCR and Western blot analysis of NOP2 mRNA and protein levels in HCC cells of different treatment groups. (C to F) Glucose uptake (C), lactate production (D), pH of the culture medium (E), and OCR (F) were tested in 3 different cell lines. (G) The ECAR was measured in 3 different cell lines using an XF Extracellular Flux Analyzer. Fig. S5. (A) Scatter plots from TCGA-LIHC database showing the correlation between the relevant genes enriched in the glycolysis signaling pathway and NOP2. (B) Scatter plots from 40 pairs of HCC tissues showing the correlation between the relevant genes enriched in the glycolysis signaling pathway and NOP2. Fig. S6. (A and B) Correlation analysis of NOP2 and c-Myc expression in HCC tissues. Fig. S7. (A and B) Representative mRNA and protein expression levels of glycolysis related genes in HCC cells subjected to different treatments. Fig. S8. (A) Glucose uptake, lactate production, the pH of the culture medium and OCR were measured in HCC cells subjected to different treatments. (B) The ECAR was measured in HCC cells subjected to different treatments. Fig. S9. (A) Tumor volume of PDX model. (B) Validation of KO efficiency of METTL5-KO in the PDX model of A. (C) Mice were monitored for weight change. Table S1. Correlation between clinicopathological features and NOP2 expression in HCC tumor tissues. Table S2. Univariate and multivariate analyses of clinicopathological characteristics, and NOP2 with overall survival. Table S3. Primer sequences and siRNAs used in this study. Table S4. Primary antibodies used in this study.
