# Supplementary material for: NOP2-mediated m5C Modification of c-Myc in an EIF3A-Dependent Manner to Reprogram Glucose Metabolism and Promote Hepatocellular Carcinoma Progression
Source: Research (Wash D C). 2023 Jun 30;6:0184. doi: 10.34133/research.0184 (PMC10313139; doi:10.34133/research.0184)
Supplement: Supplementary 1 — Fig. S1. (A) Flow cytometry results showing changes in cell cycle progression. Fig. S2. (A and B) The NOP2 overexpressing plasmid was transformed into the Hep-3B cell line. (C and D) Cell proliferation was assessed by performing CCK-8 (OD 450 nm) and colony formation. (E) Cell invasion was evaluated using Transwell invasion assay. (F) Cell migration was evaluated using wound-healing migration. Fig. S3. (A) The effect of NOP2 knockdown on sorafenib resistance. (B) The effect of NOP2 overexpression on sorafenib resistance. (C) CCK-8 assay results showing the proliferation ability of HCC cells. Fig. S4. (A and B) qRT-PCR and Western blot analysis of NOP2 mRNA and protein levels in HCC cells of different treatment groups. (C to F) Glucose uptake (C), lactate production (D), pH of the culture medium (E), and OCR (F) were tested in 3 different cell lines. (G) The ECAR was measured in 3 different cell lines using an XF Extracellular Flux Analyzer. Fig. S5. (A) Scatter plots from TCGA-LIHC database showing the correlation between the relevant genes enriched in the glycolysis signaling pathway and NOP2. (B) Scatter plots from 40 pairs of HCC tissues showing the correlation between the relevant genes enriched in the glycolysis signaling pathway and NOP2. Fig. S6. (A and B) Correlation analysis of NOP2 and c-Myc expression in HCC tissues. Fig. S7. (A and B) Representative mRNA and protein expression levels of glycolysis related genes in HCC cells subjected to different treatments. Fig. S8. (A) Glucose uptake, lactate production, the pH of the culture medium and OCR were measured in HCC cells subjected to different treatments. (B) The ECAR was measured in HCC cells subjected to different treatments. Fig. S9. (A) Tumor volume of PDX model. (B) Validation of KO efficiency of METTL5-KO in the PDX model of A. (C) Mice were monitored for weight change. Table S1. Correlation between clinicopathological features and NOP2 expression in HCC tumor tissues. Table S2. Univariate and multiv [file research.0184.f1.zip › SM Figures.docx]

Legends of Supplementary Figures

**Fig. S1.** A Flow cytometry results showing changes in cell cycle progression.

**Fig. S2.** A, B The NOP2 overexpressing plasmid was transformed into the Hep-3B cell line. C, D Cell proliferation was assessed by performing CCK-8 (OD 450 nm) and colony formation. E Cell invasion was evaluated using Transwell invasion assay. F Cell migration was evaluated using wound-healing migration.

**Fig. S3.** A The effect of NOP2 knockdown on sorafenib resistance. B The effect of NOP2 overexpression on sorafenib resistance. C CCK-8 assay results showing the proliferation ability of HCC cells.

**Fig. S4.** A, B qRT-PCR and western blot analysis of NOP2 mRNA and protein levels in HCC cells of different treatment groups. C–F Glucose uptake (C), lactate production (D), pH of the culture medium (E) and OCR (F) were tested in three different cell lines. G The ECAR was measured in three different cell lines using an XF Extracellular Flux Analyzer.

**Fig. S5.** A Scatter plots from TCGA-LIHC database showing the correlation between the relevant genes enriched in the glycolysis signalling pathway and NOP2. B Scatter plots from 40 pairs of HCC tissues showing the correlation between the relevant genes enriched in the glycolysis signaling pathway and NOP2.

**Fig. S6.** A, B Correlation analysis of NOP2 and c-Myc expression in HCC tissues.

**Fig. S7.** A, B Representative mRNA and protein expression levels of glycolysis related genes in HCC cells subjected to different treatments.

**Fig. S8.** A Glucose uptake, lactate production, the pH of the culture medium and OCR were measured in HCC cells subjected to different treatments. B The ECAR was measured in HCC cells subjected to different treatments.

**Fig. S9.** A Tumor volume of PDX model. B Validation of knockout efficiency of METTL5-KO in the PDX model of A. C Mice were monitored for weight change.

**Titles of Supplementary Tables**

**Table S1.** Correlation between clinicopathological features and NOP2 expression in HCC tumor tissues.

**Table S2.** Univariate and multivariate analyses of clinicopathological characteristics, and NOP2 with overall survival.

**Table S3.** Primer sequences and siRNAs used in this study.

**Table S4**. Primary antibodies used in this study.
