# Supplementary material for: NOP2-mediated m5C Modification of c-Myc in an EIF3A-Dependent Manner to Reprogram Glucose Metabolism and Promote Hepatocellular Carcinoma Progression
Source: Research (Wash D C). 2023 Jun 30;6:0184. doi: 10.34133/research.0184 (PMC10313139; doi:10.34133/research.0184)
Supplement: Supplementary 1 — Fig. S1. (A) Flow cytometry results showing changes in cell cycle progression. Fig. S2. (A and B) The NOP2 overexpressing plasmid was transformed into the Hep-3B cell line. (C and D) Cell proliferation was assessed by performing CCK-8 (OD 450 nm) and colony formation. (E) Cell invasion was evaluated using Transwell invasion assay. (F) Cell migration was evaluated using wound-healing migration. Fig. S3. (A) The effect of NOP2 knockdown on sorafenib resistance. (B) The effect of NOP2 overexpression on sorafenib resistance. (C) CCK-8 assay results showing the proliferation ability of HCC cells. Fig. S4. (A and B) qRT-PCR and Western blot analysis of NOP2 mRNA and protein levels in HCC cells of different treatment groups. (C to F) Glucose uptake (C), lactate production (D), pH of the culture medium (E), and OCR (F) were tested in 3 different cell lines. (G) The ECAR was measured in 3 different cell lines using an XF Extracellular Flux Analyzer. Fig. S5. (A) Scatter plots from TCGA-LIHC database showing the correlation between the relevant genes enriched in the glycolysis signaling pathway and NOP2. (B) Scatter plots from 40 pairs of HCC tissues showing the correlation between the relevant genes enriched in the glycolysis signaling pathway and NOP2. Fig. S6. (A and B) Correlation analysis of NOP2 and c-Myc expression in HCC tissues. Fig. S7. (A and B) Representative mRNA and protein expression levels of glycolysis related genes in HCC cells subjected to different treatments. Fig. S8. (A) Glucose uptake, lactate production, the pH of the culture medium and OCR were measured in HCC cells subjected to different treatments. (B) The ECAR was measured in HCC cells subjected to different treatments. Fig. S9. (A) Tumor volume of PDX model. (B) Validation of KO efficiency of METTL5-KO in the PDX model of A. (C) Mice were monitored for weight change. Table S1. Correlation between clinicopathological features and NOP2 expression in HCC tumor tissues. Table S2. Univariate and multiv [file research.0184.f1.zip › Supplementary tables S1-S4.docx]

**Supplementary Table S1** Correlation between clinicopathological features and NOP2 expression in HCC tumor tissues.

| Characteristics | Number | NOP2 expression | | P value |
| --- | --- | --- | --- | --- |
|  |  | High | Low |  |
| Gender |  |  |  | 0.289 |
| Male | 29 | 13 | 16 |  |
| Female | 11 | 7 | 4 |  |
| Age |  |  |  | 0.752 |
| ＜65 | 19 | 9 | 10 |  |
| ≥65 | 21 | 11 | 10 |  |
| HBV infection |  |  |  | 0.288 |
| No | 11 | 4 | 7 |  |
| Yes | 29 | 16 | 13 |  |
| AFP |  |  |  | 0.342 |
| ≤400 (μg/L) | 21 | 9 | 12 |  |
| ＞400 (μg/L) | 19 | 11 | 8 |  |
| Tumor size |  |  |  | ***0.025^*^*** |
| ≤5cm | 17 | 5 | 12 |  |
| ＞5cm | 23 | 15 | 8 |  |
| Lymph node metastasis |  |  |  | 0.151 |
| Absent | 35 | 16 | 19 |  |
| Present | 5 | 4 | 1 |  |
| TNM stage |  |  |  | ***0.027^*^*** |
| I-II | 19 | 6 | 13 |  |
| III-IV | 21 | 14 | 7 |  |
| BCLC stage |  |  |  | 0.185 |
| Low | 14 | 5 | 9 |  |
| High | 26 | 15 | 11 |  |
| PVTT |  |  |  | ***0.038*** |
| NO | 28 | 11 | 17 |  |
| YES | 12 | 9 | 3 |  |

*The expression of NOP2 were compared between the tumor tissue and the normal tissue. BCLC, Barcelona Clinic Liver Cancer; PVTT, portal vein tumor thrombus. Bold italics indicate statistically significant values. *P<0.05

**Supplementary Table S2** Univariate and multivariate analyses of clinicopathological characteristics, and NOP2 with overall survival.

| Characteristics | Univariate analysis | | | Multivariate analysis | | |
| --- | --- | --- | --- | --- | --- | --- |
|  | Hazard Ratio | 95% CI | P value | Hazard Ratio | 95% CI | P value |
| Gender | 1.079 | 0.446-2.609 | 0.867 |  |  |  |
| Age | 1.810 | 0.792-4.137 | 0.160 |  |  |  |
| HBV infection | 1.386 | 0.543-3.535 | 0.495 |  |  |  |
| AFP (μg/L) | 1.631 | 0.708-3.757 | 0.251 |  |  |  |
| Tumor Size (cm) | 2.821 | 1.835-4.461 | ***0.003***^*^ | 1.274 | 0.624-5.292 | 0.213 |
| BCLC staging | 2.606 | 1.048-6.482 | ***0.039***^*^ | 1.299 | 0.454-3.713 | 0.625 |
| Lymph node metastasis | 5.543 | 2.376-8.321 | ***0.001***^*^ | 2.618 | 0.877-5.920 | 0.075 |
| TNM stage | 4.790 | 2.782-6.366 | ***0.001***^*^ | 3.426 | 1.071-8.286 | ***0.040***^*^ |
| PVTT | 1.689 | 0.688-4.146 | 0.253 |  |  |  |
| NOP2 | 3.667 | 1.863-7.694 | ***0.001***^*^ | 2.919 | 1.097-6.792 | ***0.032***^*^ |

HR, Hazard ratio; CI, Confidence interval. Bold italics indicate statistically significant values. *P<0.05

**Supplementary Table S3** Primer sequences and siRNAs used in this study.

| Primer name | | Sequence (5’-3’) |
| --- | --- | --- |
| NOP2 | F | TGTCTGAGCTGGTGGAGTTCTTAG |
|  | R | ACCCCACGATTGATTAGAGCC |
| LDHA | F | CTGGGAGTTCACCCATTAAGCT |
|  | R | CAGGCACACTGGAATCTCCAT |
| c-Myc | F | GTCAAGAGGCGAACACACAAC |
|  | R | TTGGACGGACAGGATGTATGC |
| MAZ | F | TGCACAAGCCCTACAACTGCTC |
|  | R | GCACTTGTCTGACGTGACTGTTGA |
| YY1 | F | GTGCAGAATGTGGCAAAGC |
|  | R | AGGGCCTGTCTCCGGTATG |
| MAFK | F | AACACGCCGCACCTGTCGGA |
|  | R | GACTTCTGCTTCTGCAGCTC |
| TPI1 | F | AGTGACTAATGGGGCTTTTACTG |
|  | R | GCCCAATCAGCTCATCTGACTC |
| ENO1 | F | ATGTCTATTCTCAAGATCCATGCCAGG |
|  | R | CTACTTGGCCAAGGGGTTTCTGAAG |
| PKM2 | F | ATGTCGAAGCCCCATAGTGAA |
|  | R | TGGGTGGTGAATCAATGTCCA |
| β-actin | F | GGCATGGGTCAGAAGGATT |
|  | R | CACACGCAGCTCATTGTAGA |
| Control siRNA |  | UUCUCCGAACGUGUCACGUTT |
|  |  | ACGUGACACGUUCGGAGAATT |
| NOP2 siRNA1 |  | CCCACAAUAUGGAUGGGUUTT |
|  |  | AACCCAUCCAUAUUGUGGGTT |
| NOP2 siRNA2 |  | GCAACGAUCACCUAAAUUATT |
|  |  | UAAUUUAGGUGAUCGUUGCTT |
| NOP2 sgRNA | | TCGGACACATTAACCCGCATTC |

**Supplementary Table S4** Primary antibodies used in this study

| Antigens | Manufacturer | Catalog Number | Application |
| --- | --- | --- | --- |
| NOP2 | Abcam | Ab271075 | 1:1000 for WB;  1:200 for IHC; |
|  |  |  | 1:1 for IP |
| LDHA | Proteintech | 21799-1-AP | 1:1000 for WB;  1:200 for IHC |
|  |  |  |  |
| β-actin | Proteintech | 66009-1-Ig | 1:1000 for WB |
|  |  |  |  |
| TPI1 | Proteintech | 10713-1-AP | 1:10000 for WB;  1:200 for IHC |
| ENO1 | Proteintech | 11204-1-AP | 1:1000 for WB;  1:200 for IHC |
| PKM2 | Proteintech | 60268-1-Ig | 1:1000 for WB;  1:200 for IHC |
| c-Myc | Abcam | ab32072 | 1:1000 for WB;  1:500 for IHC |
| MAZ | Proteintech | 21068-1-AP | 1:1000 for WB |
| Caspase-3 | CST | #9662 | 1:10000 for WB |
| Caspase-7 | CST | # 9494S | 1:10000 for WB |
| Caspase-9 | CST | # 9508S | 1:10000 for WB |
| IgG | Servicebio | GB23303 | IP |
| IgG | Servicebio | GB23301 | IP |
| HRP | IPKine | A25222 | IP |
